# National Evaluation of HIV Service Resource Allocation in Tanzania

**DOI:** 10.1007/s10461-023-04065-5

**Published:** 2023-05-05

**Authors:** Ryan K. McBain, Monica Jordan, Carlyn Mann, George M. Ruhago, Bryant Lee, Steven Forsythe, Kaylee Stewart, Jessica Brown, Allyala Nandakumar

**Affiliations:** 1 Center for Integration Science, Brigham and Women’s Hospital, Boston, MA 02115 USA; 2 Institute for Global Health and Development, Brandeis University, Waltham, MA USA; 3 USAID, Washington, DC USA; 4 Muhimbili University of Health and Allied Sciences, Dar es Salaam, Tanzania; 5 The Palladium Group, Washington, DC USA; 6 Avenir Health, Glastonbury, CT USA

**Keywords:** Time-driven activity-based costing, Cost analysis, Equity, Health systems strengthening, Resource allocation

## Abstract

Using time-driven activity-based costing (TDABC), we examined resource allocation and costs for HIV services throughout Tanzania at patient and facility levels. This national, cross-sectional analysis of 22 health facilities quantified costs and resources associated with 886 patients receiving care for five HIV services: antiretroviral therapy, prevention of mother-to-child transmission, HIV testing and counseling, voluntary medical male circumcision, and pre-exposure prophylaxis. We also documented total provider-patient interaction time, the cost of services with and without inclusion of consumables, and conducted fixed-effects multivariable regression analyses to examine patient- and facility-level correlates of costs and provider-patient time. Findings showed that resources and costs for HIV care varied significantly throughout Tanzania, including as a function of patient- and facility-level characteristics. While some variation may be preferable (e.g., needier patients received more resources), other areas suggested a lack of equity (e.g., wealthier patients received more provider time) and presented opportunities to optimize care delivery protocols.

## Introduction

In Tanzania, HIV/AIDS is the third largest contributor to morbidity and mortality [1]—affecting more than 1.7 million people and accounting for approximately 27,000 deaths per year [2]. Considerable resources over the past two decades have been dedicated to tackling this disease. While the Government of Tanzania (GoT) allocates approximately US$54 million per year of its own resources, it receives almost $600 million in financial support from PEPFAR and the Global Fund [3]. This has translated to significant progress in meeting 90–90–90 targets [4]. In 2020 alone, nearly 170,000 new adults and children were placed on antiretroviral therapy (ART), and more than 100,000 people with HIV were newly identified through index testing [5]. Over 1.4 million individuals on ART have suppressed viral loads [6].

As these milestones accumulate, important questions remain about how HIV services will continue to be financed and how resources should be configured most effectively and equitably [3]. Beginning in the 2017–2018 funding cycle, the Global Fund shifted to a co-financing model in which the GoT has assumed incrementally greater fiscal responsibility, year-over-year, for supporting HIV services [7]. At the same time, the county launched a differentiated care model, through which persons living with HIV/AIDS with a suppressed viral load receive streamlined services (e.g., multi-month dispensing of antiretrovirals), while those who have not achieved viral suppression receive more frequent and intensive services [8]. At the same time, health clinics in Tanzania have begun to pilot integrated care and management for HIV and non-communicable diseases like hypertension and diabetes [9].

Whether and to what extent changes in financing and care delivery models have impacted service delivery on the ground remains an open question. Anticipated changes may be influenced by an array of factors such as the quality and intensity of training and mentorship, degree of accountability, staffing levels, and availability of equipment and medicines [10, 11]. Likewise, financial resources intended to reach clinics and patients may be depleted throughout administrative bureaucracies [12].

To investigate the arrangement of resources and corresponding HIV service delivery at facility and patient levels requires a model that is nuanced in its ability to capture detailed information and flexible in its application across a wide array of settings throughout Tanzania. Time-driven activity-based costing (TDABC) is one such model: it employs direct observation of patients as they move through the healthcare system to quantify the resources that each patient consumes [13]. This includes: where patients go, whom they see, what services they receive, what laboratory tests are ordered, and what medications are prescribed and dispensed [14]. The result is a detailed inventory of patient-level information on care delivery patterns and resource consumption.

TDABC underpins a new, multi-country initiative to quantify HIV-related resources and expenditures in sub-Saharan Africa—including Tanzania. The initiative, known as Activity-based Costing and Management (ABC/M), aims to align and optimize country governments’ and global institutions' investments for HIV/AIDS. In this study, we apply TDABC to investigate resource allocation for HIV services in Tanzania from two complementary perspectives: first, overall resource consumption in terms of costs and cost drivers; second, equity in resource allocation, based on patient- and facility-level characteristics associated with resource consumption. This information will be used by the GoT, implementation partners and funders to understand the state of HIV services throughout the country, and to optimize services based on this information.

## Methods

### Setting and Sample Characteristics

The ABC/M Initiative is currently being implemented in six African countries, including Tanzania, and is led by the Office of the U.S. Global AIDS Coordinator and USAID. While its main emphasis pertains to aligning and optimizing bilateral and multilateral investments for HIV/AIDS, it also aims to establish a sustainable framework for routinely quantifying resources and costs directed to HIV services. The initiative is supported by a global coalition comprising The Global Fund, the Joint United Nations Program on HIV/AIDS (UNAIDS), the U.S. Center for Disease Control and Prevention (CDC), and the U.S. Treasury. Tanzania is the first of six countries to have completed baseline data collection, with facility-level information representing the focus of this manuscript.

Data collection concentrated on six regions/four geographic zones of the country with the highest HIV prevalence. Dar es Salaam was also selected due to its unique characteristics, including that one-sixth of all patients in the country receiving ART are located there, and it is often the first area of the country to pilot newer models of service delivery, such as six-month ART dispensing. Among these regions, 22 facilities were purposively selected to ensure heterogeneity across an array of characteristics, including: geography (urban vs. rural), facility type (district hospital, health center, dispensary), funding support from PEPFAR (yes vs. no), sector (public vs. private/NGO), and HIV patient cohort volume [low (20–249 patients), medium (250–1249 patients), high (1250+ patients)]. See Table 1 for details.

**Table 1:** Characteristics of participating HIV facilities

Facility name	Region	Facility type	Urbanicity	Public/private	PEPFAR support	HIV client volume
*Dispensaries*						
Boko Dispensary	Dar es Salaam	Dispensary	Urban	Public	Yes	Low
Mahaha Dispensary	Mwanza	Dispensary	Rural	Public	Yes	Medium
Luhanga Dispensary	Mwanza	Dispensary	Urban	Public	Yes	Low
Mwanzugi Dispensary	Tabora	Dispensary	Rural	Private/FBO	Yes	Medium
Mwisole Dispensary	Tabora	Dispensary	Rural	Public	No	Low
*Health centers*						
Bunazi Health Centre	Kagera	Health Centre	Rural	Public	Yes	High
Chipanga Health Centre	Dodoma	Health Centre	Rural	Public	Yes	Medium
Igawilo Health Centre	Mbeya	Health Centre	Rural	Public	Yes	Medium
Inyala Health Centre	Mbeya	Health Centre	Rural	Public	Yes	Medium
Kiwanja Health Centre	Mbeya	Health Centre	Urban	Public	Yes	High
Lupembe Health Centre	Njombe	Health Centre	Rural	Public	Yes	Medium
Magomeni Health Centre	Dar es Salaam	Health Centre	Urban	Public	Yes	High
Makole Health Centre	Dodoma	Health Centre	Urban	Public	Yes	High
Njombe Health Centre	Njombe	Health Centre	Urban	Public	Yes	High
Upuge Health Centre	Tabora	Health Centre	Rural	Public	Yes	Low
*Hospitals*						
Biharamulo District Hospital	Kagera	Hospital	Rural	Public	Yes	High
Ilembula Hospital	Njombe	Hospital	Rural	Private/FBO	Yes	High
Mbagala District Hospital	Dar es Salaam	Hospital	Urban	Public	Yes	High
Misungwi District Hospital	Mwanza	Hospital	Rural	Public	Yes	High
Mvumi Mission Hospital	Dodoma	Hospital	Rural	Private/FBO	Yes	Medium
Nzega District Hospital	Tabora	Hospital	Rural	Public	Yes	High
Tukuyu District Hospital	Mbeya	Hospital	Rural	Public	Yes	High

HIV client volume was defined as: low 20–249 clients with HIV per month), medium (250–1249 patients, high (1250+ patients)

*FBO* faith-based organization, *GoT* Government of Tanzania, *PEPFAR*, Presidents Emergency Plan for AIDS Relief. PEPFAR support reflects financial contributions from PEPFAR

All facilities provided a comprehensive array of HIV services that included routine ART, HIV testing and counseling (HTC), prevention of mother-to-child transmission (PMTCT) services, voluntary medical male circumcision (VMMC), and pre-exposure prophylaxis (PrEP) and were fully operational for at least 2 years. For ART, we distinguished between “stable patients,” who are virally suppressed and receive ART services once per quarter, from “unstable patients,” who are not virally suppressed and receive ART services once per month until viral suppression is achieved. We also note that specific services, like PMTCT, encompassed constituent elements from other services, like ART provision. For the purposes of this analysis, the distinguishing feature of each service pertained to the chief complaint (or reason for visit) of a client and the corresponding location of service. For example, PMTCT is distinct from ART insofar as it is a programmatic service taking place in the antenatal and postnatal care context, which targets prevention of vertical transmission through ART and other service components such as education about best practices for breastfeeding.

Individuals were eligible to participate if they were aged 18+ and were accessing HIV services (listed above) at one of the 22 participating facilities between September 2 and October 16, 2020. Eligible participants were identified upon registration at the facility, at which point the individual was informed about the study objectives and presented with a study consent form. Those who consented were assigned a unique identifier for tracking purposes. Institutional review approval was received from institutions in Tanzania (Institute for Medical Research; Muhimbili University of Health and Allied Sciences) and the United States (Health Media Lab).

### Procedures

TDABC. A data collection team, led by the Muhimbili University of Health and Allied Sciences, executed TDABC procedures in accordance with best practices [15]. Briefly, these steps comprised:

1. *Select medical services and populations*: As stated above, the scope of our analysis was limited to five service lines (ART, HTC, PMTCT, VMMC, and PrEP) among adult clients who were attending care at one of 22 facilities selected.
2. *Define the care delivery value chain*: Team members developed a prescriptive inventory of potential activities and resources allocated to clients at each facility—to ensure that these would be subsequently reviewed during direct observation of patients and staff. Inventories were developed based on key informant interviews with facilities-in-charge.
3. *Obtain time estimates for activities*: Lay health workers, trained as data collectors, were responsible for direct observation of patients at each step in the care delivery process. Data collectors were equipped with stopwatches and recorded provider time with patients. Separately, data collectors interviewed providers to gather information on provider tasks performed that did not involve direct patient-provider interactions. Three mechanisms were put in place to ensure data quality and consistency. First, upon completion of training, data collectors completed a post-training test. Second, data collectors were supervised by lead data officers, who performed random spot checks on their activities. Third, lead data officers aggregated and reviewed information across data collectors on a weekly basis to identify outliers and anomalies, and to provide feedback for course corrections when necessary.
4. *Develop process maps for medical services*: Process maps were inductively generated based on a synthesis of information gathered by lay health workers [16]. Process maps conveyed the order, location, and frequency of steps in the care process, as well as providers engaged, and consumables utilized. Maps were assembled by lead data officers on the project, after reviewing and synthesizing data from direct observations.
5. *Obtain time estimates for activities*: Lay health workers, trained as data collectors, were responsible for direct observation of patients at each step in the care delivery process. Data collectors were equipped with stopwatches and recorded provider time with patients. Separately, data collectors interviewed providers to gather information on provider tasks performed that did not involve direct patient-provider interactions.
6. *Gather cost information*: Cost estimates were gathered from facility ledgers, electronic financial systems, and price lists—including The Global Fund’s pooled procurement drug list. A conversion rate of 2,300 Tanzanian Shillings to 1 US Dollar was used throughout. Salaries were self-reported and included fringe benefits. Equipment costs were annualized based on linear depreciation over estimated useful lifespan. Indirect costs included support staff and other overhead expenses needed to provide services that were not directly related to HIV service provision. Note that we were concerned with costs at the facility level; we did not include costs above the facility level, such as those corresponding to supply chain delivery of medications to facilities.
7. *Estimate practical capacity of resources*: Practical capacity is defined as the availability of a resource (e.g., personnel, space) over a fixed time interval for resource consumption by a patient [13]. For the purposes of this project, we operationalized practical capacity as minutes per year that each resource was available to patients. We gathered this information through key informant interviews with each type of cadre present at facilities. Based on this, we calculated capacity cost rates, which divide the total cost of a resource by its practical capacity.
8. *Calculate patient-level costs for medical services*: We calculated patient-level costs by multiplying capacity cost rates by the duration of time (in minutes) that each patient consumed resources, in addition to price and quantity of consumables, aggregated across all steps over the course of care delivery. Based on the continuous nature of ART services, cost estimates were annualized for both stable and unstable clients. All other services were interpreted as one-time encountered, and cost estimates therefore reflected the total cost of care during a single facility encounter.

Upon conclusion of participants’ medical visits, they were invited to complete a brief client exit interview. These interviews gathered demographic and socioeconomic information. Due to COVID-19, we made two adjustments to the implementation process. First, supervisors administered daily health checks to team members, isolating anyone feeling sick or displaying COVID-19 symptoms. Second, research teams were required to practice physical distancing, wear personal protective equipment, and maintain strict protocols for routine cleaning and disinfection of equipment.

### Statistical Analysis

We conducted statistical analyses in three steps. First, we performed descriptive analyses to characterize the frequency, quantity, and distribution of costs and resources for HIV service lines. This included, for example, inspecting the range of costs for individual service lines across facilities, as well as the most-utilized human resources in terms of expenditure per visit. All analyses were performed using Stata 17.0 [17].

Second, we conducted fixed-effects multivariable linear regression analyses to investigate whether patient-level demographic characteristics predicted differences in the magnitude of resources allocated to patients in terms of (i) total visit duration and (ii) total visit costs, with and without inclusion of consumables. The analysis included an indicator variable for service type (e.g., ART = 1, HTC = 2, PMTCT = 3, VMMC = 4, and PrEP = 5) in order to account for differences in cost and visit duration resulting from this aspect of service delivery. In terms of patient characteristics of interest, we examined: gender (female, male), age group (18–30, 31–50, 51–70, 71+), marital status (married, unmarried), years of education, existence of comorbid health conditions (yes, no), and household wealth–as indicated by a household asset index comprising information on whether the household possessed certain items (e.g. mobile phone, refrigerator, television) or reflected certain characteristics (e.g. quality of flooring and roofing). Patient characteristics were chosen based on prior literature indicating that HIV service quality and costs, as well as health-seeking behavior, sometimes vary by these characteristics [18–21].

The process for deriving the household asset index was based on the standard convention of conducting principal component analysis and specifying a single factor [22]. Multivariable regression models also contained fixed effects to account for facility characteristics, including: region, facility type, facility rurality, funder, and HIV patient volume. These characteristics were included because they served as inputs to the sampling frame, and we had a priori expectations that differences may be observed along these axes. We examined three models, one for each outcome: patient-provider duration, cost without consumables, cost with consumables. Analyses were based on a two-tailed alpha level of 0.05.

## Results

### Descriptive Analysis

Out of the 886 participants in the study, 69% were female and 31% male, although this varied considerably according to service line (see Table 2). Average age of participants was 36.6 (SD: 12.2), while average years of education was 2.9 (SD: 1.3). Participants were low-income, as indicated by household possessions: only 23% of participants had electricity, 37% owned a mobile phone, and 3% owned a refrigerator. Almost half of the sample was married (49%).

**Table 2:** Study participant characteristics—by service line

Service line	Sample size	Female (%)	Age (SD)	Married (%)	Years of edu. (SD)	Comorbid conditions (%)
ART, stable	240	147 (61%)	43.3 (11.2)	104 (43%)	2.9 (1.3)	38 (16%)
ART, unstable	214	145 (68%)	39.4 (11.9)	85 (40%)	2.7 (1.2)	38 (19%)
HTC	198	104 (53%)	33.5 (13.2)	101 (51%)	3.2 (1.4)	18 (9%)
PMTCT	200	200 (100%)	30.0 (6.6)	128 (64%)	2.9 (1.1)	4 (3%)
PrEP	15	13 (87%)	26.9 (6.3)	7 (47%)	3.3 (1.0)	0 (0%)
VMMC	19	0 (0%)	29.1 (10.9)	11 (58%)	2.1 (1.1)	0 (0%)
Total	886	607 (69%)	36.6 (12.2)	436 (49%)	2.9 (1.3)	98 (12%)

“Comorbid condition” represents any other medical diagnosis, including infectious and non-communicable diseases, recorded during the patient encounter

*ART* antiretroviral therapy, *HTC* HIV testing and counseling, *PMTCT* prevention of mother-to-child transmission, *PrEP* pre-exposure prophylaxis, *SD* standard deviation, *VMMC* voluntary medical male circumcision

Table 3 provides a breakdown of service-level costs per visit according to four cost categories: human resources, space/equipment, indirects, and consumables. We found that VMMC was the highest cost per visit ($28.00), followed by ART for virally suppressed individuals ($22.72), PMTCT ($22.12), ART for individuals who are not virally suppressed ($14.86), PrEP ($6.77) and HTC ($3.67). Given the recurrent nature of ART services across the lifespan for persons living with HIV, Table 3 presents annualized costs for these: an average of $90.88 for virally suppressed individuals (stable), and $178.32 for individuals who were not virally suppressed (unstable). Within each service line, consumables–comprising medications, labs, and items such as syringes and gauze–accounted for a large preponderance of costs ranging from 61 to 95%. In all instances except HTC, consumable costs were dominated by the cost of medications. This was followed by human resources (3% to 22%), indirects (2% to 14%) and space/equipment (< 1% to 3%).

**Table 3:** Service line costs per visit and variation across facilities

Service line	Mean cost (SD)	% of total cost	IQR
*ART—stable*^b^	$90.88 ($33.84)	100.0	$72.04, $83.44
Human resources	$3.00 ($3.24)	3.3	$1.32, $3.52
Space/equipment	$0.40 ($0.52)	0.4	$0.16, $0.48
Indirects	$1.56 ($1.88)	1.7	$0.28, $2.04
Consumables	$85.96 ($33.12)	94.6	na^a^
*ART—unstable*^b^	$178.32 ($119.76)	100.0	$112.08, $151.44
Human resources	$9.72 ($9.00)	5.5	$4.80, $11.16
Space/equipment	$1.44 ($1.68)	0.8	$0.48, $1.80
Indirects	$4.32 ($5.16)	2.4	$0.96, $5.76
Consumables	$162.72 ($116.76)	91.3	na^a^
*HIV testing/counseling (HTC)*	$3.67 ($1.78)	100.0	$2.32, $4.57
Human resources	$0.82 ($0.68)	22.3	$0.35, $1.05
Space/equipment	$0.11 ($0.22)	3.0	$0.03, $0.11
Indirects	$0.50 ($0.52)	13.6	$0.10, $0.78
Consumables	$2.24 ($1.05)	61.0	$1.43, $3.06
*PMTCT*	$22.12 ($20.21)	100.0	$9.26, $46.34
Human resources	$1.16 ($0.91)	5.2	$0.52, $1.48
Space/equipment	$0.13 ($0.14)	0.6	$0.04, $0.17
Indirects	$0.44 ($0.43)	2.0	$0.10, $0.62
Consumables	$20.40 ($19.84)	92.2	$7.82, $43.58
*PrEP*	$6.77 ($1.64)	100.0	$5.50, $8.23
Human resources	$0.59 ($0.72)	8.7	$0.09, $1.04
Space/equipment	$0.04 ($0.03)	0.6	$0.01, $0.05
Indirects	$0.15 ($0.35)	2.2	$0.00, $0.17
Consumables	$5.99 ($0.79)	88.5	$5.40, $6.98
*VMMC*	$28.00 ($9.01)	100.0	$23.64 $33.54
Human resources	$3.97 ($1.73)	14.2	$2.58, $5.05
Space/equipment	$0.43 ($0.22)	1.5	$0.14, $0.59
Indirects	$0.91 ($1.09)	3.3	$0.17, $2.22
Consumables	$22.69 ($7.30)	81.0	$20.80. $27.04

*ART* antiretroviral therapy, *HTC* HIV testing and counseling, *IQR* interquartile range, *PMTCT* prevention of mother-to-child transmission, *PrEP* pre-exposure prophylaxis, *SD* standard deviation, *VMMC* voluntary medical male circumcision

^a^IQR was not applicable (na) for consumables because the cost values were uniform across facilities

^b^Annualized cost presented for ART-Stable and ART-Unstable. All other conditions presented as cost per encounter

Other than consumables, which were considered a fixed cost within each service line, we observed significant heterogeneity in total expenditure across facilities. For example, the interquartile range (IQR) for the cost of a visit for PMTCT was $9.26 to $46.34 (a five-fold difference). Other service lines, such as ART for stable patients, were more consistent–with an IQR of $72.04 to $83.44–primarily because the medications dispensed were standardized.

### Multivariable Regression Analysis

**Fig. 1:**
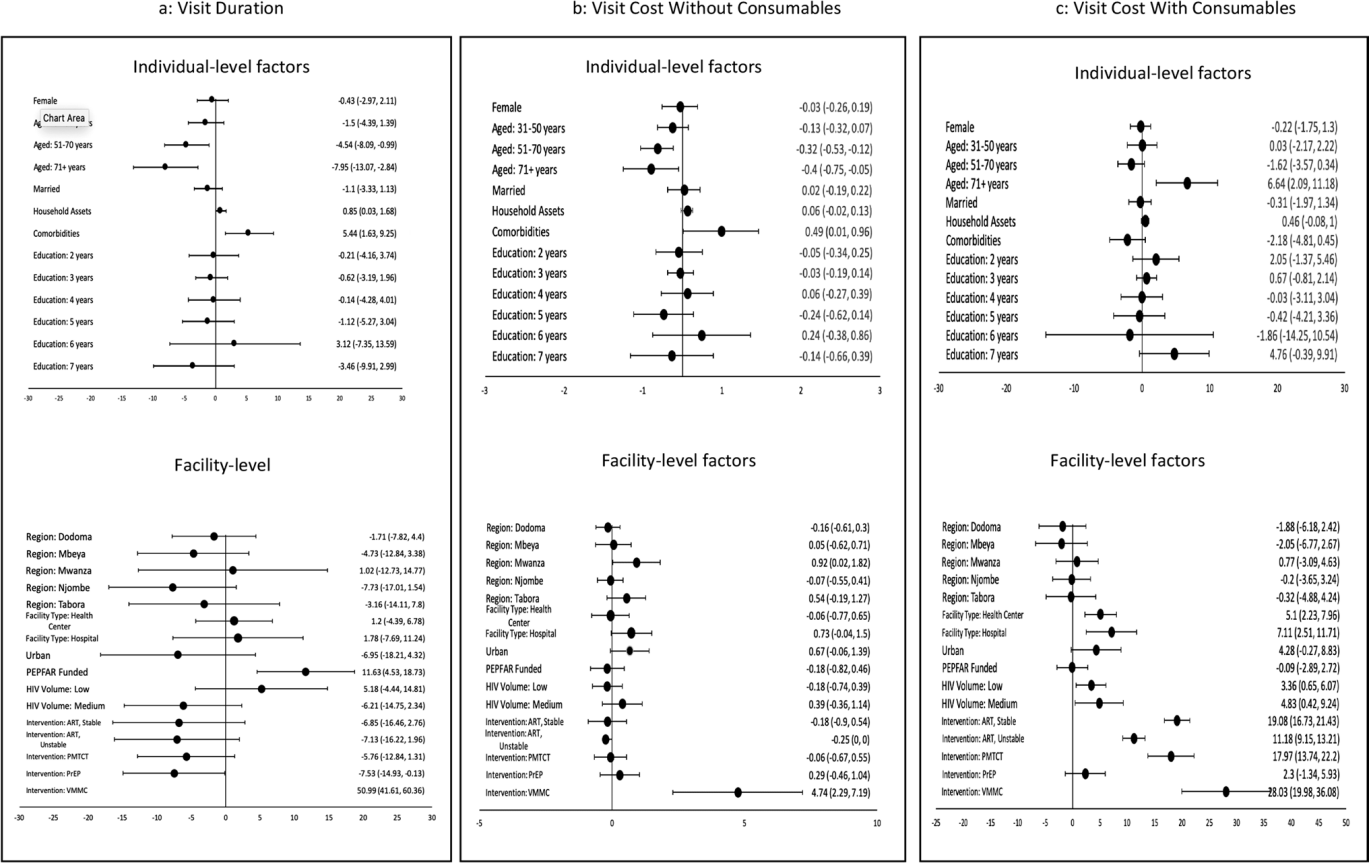
Visit duration and cost with and without consumables. Measures of effect size from multivariable regression models. **a** Differences in visit duration, in minutes, as well as 95% confidence intervals. For age categories, reference group is adults ages 18–30; for marital status, reference group is unmarried; for household assets, comparator is a one-unit difference in magnitude of household assets; for comorbidities, reference group is adults with no comorbid conditions; for education, reference group is 0–1 years of education; for region, reference region is Dar Es Salaam; for facility type, reference type is dispensaries; for funding, reference funder is the Government of Tanzania; for HIV volume, reference level was ‘high’; and for intervention, reference intervention was HIV testing and counseling. For **b**, **c**, model coefficients are expressed in US dollars

Multivariable regression analyses examined patient-level and facility-level characteristics that, in conjunction, predicted higher/lower levels of resource allocation for individual encounters. Figure 1a–c provide graphical representations of the results and corresponding 95% confidence intervals across all service lines. As shown in Fig. 1a, we observed that statistically significant predictors of longer provider-patient interactions included being under 30 years old (β = 8.0 additional minutes, 95% CI 2.8, 13.1; p = 0.004), having greater household assets (β = 0.9, 95% CI 0.0, 1.7; p = 0.04), and having comorbidities (β = 5.4, 95% CI 1.6, 9.3; p = 0.01). At the facility level, this included being funded through PEPFAR compared to the GoT (β = 11.6, 95% CI 4.5, 18.7; p = 0.003). Additionally, compared to HTC, VMMC took 51 more minutes (β = 51.0, 95% CI: 41.6, 60.4; p < 0.001) while PrEP took 7.5 fewer minutes (β = − 7.5, 95% CI − 14.9, − 0.1; p < 0.05). The duration of other service lines was not statistically different from HTC. The R-squared value for this model was 0.34.

A handful of these findings were replicated when inspecting visit cost without inclusion of consumables (Fig. 1b). Specifically, visits with younger adults cost more compared to older adults (β = $0.40 more, 95% CI $0.05, $0.75; p = 0.03), as did visits for individuals with versus without comorbidities (β = $0.49, 95% CI $0.01, $0.96; p = 0.04). Additionally, services in Mwanza District were (on average) more expensive (β = $0.92, 95% CI $0.02, $1.82; p < 0.05), as was VMMC as a service line compared to HTC (β = $4.74, 95% CI $2.29, $7.19; p = 0.001). The R-squared value for this model was 0.39.

Once consumables were factored into total visit cost (Fig. 1c), we found that most differences were no longer significant. Instead, we observed that older individuals above age 70 (β = $6.64, 95% CI $2.09, $11.18; p = 0.01) were more costly compared to younger individuals–presumably because they were more likely to receive medications and lab tests. We also found that health centers (β = $5.10, 95% CI $2.23, $7.96; p < 0.001) and hospitals (β = $7.11, 95% CI $2.51, $11.71; p < 0.001) cost more compared to dispensaries, and that ART for stable patients (β = $19.08, 95% CI $16.73, $21.43; p < 0.001), ART for unstable patients (β = $11.18, 95% CI $9.15, $13.21; p < 0.001), PMTCT (β = $11.97, 95% CI $13.74, $22.20; p < 0.001) and VMMC (β = 28.03, 95% CI $19.98, $36.08; p < 0.001) were more expensive than HTC–again, likely as a function of more medications prescribed and labs ordered. Lastly, facilities with either a low (β = $3.36, 95% CI $0.65, $6.07; p = 0.02) or moderate volume (β = $4.83, 95% CI $0.42, $9.24; p = 0.03) of HIV patients generated a higher cost, compared to those with a larger patient volume. The R-squared value for this model was 0.35.

## Discussion

This manuscript provides a detailed presentation of patient- and facility-level service costs for providing HIV care between September 2 and October 16, 2020 among more than 800 patients at 22 health care facilities throughout Tanzania. We found that the average cost of service provision ranged from $3.67 for an HTC visit to $28.00 for VMMC. The annualized, recurrent cost of ART for stable patients averaged $90.88; for unstable patients this was $178.32. A large preponderance of these costs was driven by consumables that included medications and labs (61–91% of total costs), followed by human resources (3–22%), indirect costs (2–14%), and space and equipment (1–2%). Our observed pattern of costs is consistent with prior research, including in Tanzania [23–25]. We also observed that costs varied widely across individuals, facilities, and service lines, and that these were partially explained by patient- and facility-level characteristics.

In several instances, variation in time and resources appeared to signal a degree of vertical equity within Tanzania’s health system, meaning individuals with greater needs appeared to receive greater resources [26]. For example, older patients–who are typically more frail–generated a higher cost than younger patients when this cost accounted for consumables. Additionally, we saw a pattern by which providers spent more time with patients at health centers and hospitals compared to dispensaries, consonant with an expectation that individuals visiting higher levels of care have more complex symptom profiles that merit closer attention [27, 28]. Further along these lines, we observed that patients with comorbidities received more time with providers compared to those without comorbidities. Collectively, these findings signal that the healthcare delivery system is functioning in a way that is adapted to the presenting needs of patients.

We also documented a countervailing pattern of associations that could be interpreted as a signal of compromised equity. For instance, wealthier individuals appeared to spend more time with providers. On the one hand, given the prevalence of informal payments, it could be the case that wealthier patients are willing or able to pay more into the system to receive preferential treatment [29–31]; on the other hand, wealthier patients could be asking more questions and demanding more services, compared to less wealthy individuals. Prior research has documented both patterns of behavior [32–34]. Likewise, younger individuals–despite receiving fewer consumables such as medications–spent more time with providers and other staff over the course of their visits. We also found that patient visits were longer at PEPFAR-funded facilities overall, and more consumables appeared to be allocated to patients in Dar Es Salaam compared to other regions of the country such as Mwanza and Tabora. In settings such as Ethiopia and Zambia, researchers have found that facilities with external financial support tend to be better resourced in terms of staffing, equipment, and medications [35, 36]. This may also be the case in Tanzania, though measurement from a single non-PEPFAR supported facility limits our ability to infer beyond the sample. As a population-dense capital city, it is also unsurprising that facilities in Dar Es Salaam have greater access to consumables, compared to other population centers.

Taken together, these findings provide the GoT with the opportunity to review occurrences of inequalities in allocation of resources throughout the county, at patient and facility levels, and to finetune investments. Initiatives prospectively tracking resource allocation over several years are underway in countries such as Namibia and South Africa, as well as broader global efforts such as the Resource Tracking for HIV Prevention Research and Development Working Group [37–39]. These may represent relevant models to emulate. Additionally, given that consumables account for most expenditures associated with HIV service delivery, it would behoove the GoT to bolster supply chain systems. Past analyses in Tanzania have revealed challenges with consistent and adequate stocking of essential medications [40]. While current supplies are often subsidized by bilateral and multilateral institutions such as PEPFAR and the Global Fund, these investments may be tapered over time. In this case, the GoT may have an even stronger motivation for shoring up its supply chain.

We note several study limitations. First, for the purposes of this investigation, consumables were interpreted as a fixed cost: meaning, if a patient received medications or lab tests, the corresponding services received and–by extension–costs were considered invariant. Given the sizable contribution of consumables to total service costs, future data collection efforts should separately catalog the medications received and labs performed for individual patients. Second, our total sample size (n = 886) reflects patients for whom all sociodemographic characteristics were appropriately documented. A small minority of patients were excluded due to missing information. While we expect that these data are missing at random, it is not possible to verify this. Third, for PrEP and VMMC, we collected data on fewer than 20 patients. While this was a function of the frequency that these services were performed, it would be ideal to have a larger sample size to have more certainty of cost estimates and resources allocated. Lastly, our analysis did not incorporate program management, community-based and above-facility expenditures. Our analysis was narrowly concerned with facility-level operations and the resources allocated to individual patients at each facility.

## Conclusions

This study provides an in-depth analysis of resource allocation and costs associated with HIV service delivery throughout Tanzania. In addition to cataloging cost estimates, cost variation, and cost drivers, we also examined patient- and facility-level characteristics that correlated with provider-patient interaction duration and total costs. This represented a data-driven approach to evaluating equity in the context of service delivery, and identified certain strengths (e.g., older and sicker patients appeared to receive more resources) as well as weaknesses (e.g. wealthier patients and those attending PEPFAR-funded facilities received more attention). These findings should set the stage for dialog about best practices and help guide strategic planning efforts.

## Data Availability

Data analyzed during this study are not publicly available.

## Code Availability

Analytic code available upon request from the corresponding author.
